# Inter-physician variability in strategies linked to treatment limitations after severe traumatic brain injury; proactivity or wait-and-see

**DOI:** 10.1186/s12910-021-00612-8

**Published:** 2021-04-13

**Authors:** Annette Robertsen, Eirik Helseth, Reidun Førde

**Affiliations:** 1 Division of Emergencies and Critical Care, Department of Research and Development, Oslo University Hospital, Oslo, Norway; 2 Institute of Clinical Medicine, University of Oslo, Oslo, Norway; 3 Department of Neurosurgery, Oslo University Hospital, Oslo, Norway; 4 Centre of Medical Ethics, University of Oslo, Oslo, Norway

**Keywords:** Decision-making, Ethics, Severe traumatic brain injury, Disorders of consciousness, Futile treatment

## Abstract

**Background:**

Prognostic uncertainty is a challenge for physicians in the neuro intensive care field. Questions about whether continued life-sustaining treatment is in a patient’s best interests arise in different phases after a severe traumatic brain injury. In-depth information about how physicians deal with ethical issues in different contexts is lacking. The purpose of this study was to seek insight into clinicians’ strategies concerning unresolved prognostic uncertainty and their ethical reasoning on the issue of limitation of life-sustaining treatment in patients with minimal or no signs of neurological improvement after severe traumatic brain injury in the later trauma hospital phase.

**Methods:**

Interviews with 18 physicians working in a neurointensive care unit in a large Norwegian trauma hospital, followed by a qualitative thematic analysis focused on physicians’ strategies related to treatment-limiting decision-making.

**Results:**

A divide between proactive and wait-and-see strategies emerged. Notwithstanding the hospital’s strong team culture, inter-physician variability with regard to ethical reasoning and preferred strategies was exposed. All the physicians emphasized the importance of team—family interactions. Nevertheless, their strategies differed: (1) *The proactive physicians* were open to consider limitations of life-sustaining treatment when the prognosis was grim. They initiated ethical discussions, took leadership in clarification and deliberation processes regarding goals and options, saw themselves as guides for the families and believed in the necessity to prepare families for both best-case and worst-case scenarios. (2) *The “wait-and-see” physicians* preferred open-ended treatment (no limitations). Neurologically injured patients need time to uncover their true recovery potential, they argued. They often avoided talking to the family about dying or other worst-case scenarios during this phase.

**Conclusions:**

Depending on the individual physician in charge, ethical issues may rest unresolved or not addressed in the later trauma hospital phase. Nevertheless, team collaboration serves to mitigate inter-physician variability. There are problems and pitfalls to be aware of related to both proactive and wait-and-see approaches. The timing of best-interest discussions and treatment-limiting decisions remain challenging after severe traumatic brain injury. Routines for timely and open discussions with families about the range of ethically reasonable options need to be strengthened.

## Background

Variability in treatment-limiting practices after severe traumatic brain injury (sTBI) is a concern [1–3]. Although guidelines on ethical and communicational aspects are developed, life-death decisions by physicians when patients lack capacity are prone to be influenced by bias, emotions, personality and culturally bound ways of thinking, especially in the context of multi-level uncertainties [4–7]. In-depth information about how physicians handle ethical dilemmas in different phases after sTBI is lacking.

The recent Choosing Wisely Campaign intends to improve patient-centered care and seek to avoid futile or unwanted interventions [8]. For critical care one of the key recommendations is: “Do not continue life-sustaining treatments (LST) for patients at high risk of death or severely impaired functional recovery without offering patients and their families the alternative of care focused entirely on comfort” [8].

A common understanding of key concepts is a prerequisite for successful communication. The term “*futile*” is a concept often used by physicians to justify limitation of LST and refers to cases where intended physiological goals are not possible to achieve [9, 10]. “*Potentially inappropriate treatment*” (PIT) refers to more complex and ethically challenging cases where although certain goals might be attainable, treatment may not be in the patient’s best interest. Both concepts lack meaning unless the goals of treatment are specified. In the early phases after an injury, treatment may be given to obtain short-term goals such as avoiding imminent death, obtaining physiological stabilization, optimizing conditions for potential recovery, minimalizing secondary injuries or complications, giving sufficient time to gather a firm decisional ground including time to follow the individual patient’s response to treatment efforts. In the later trauma hospital phase the long-term goal gradually play the most important role. An open deliberation process weighing medical facts and clarifying goals, interests and values may reduce unresolved doubt and increase the quality of the decision made [9, 11].

Although sTBI causes a high risk of death or severe neurological impairment, some patients do recover far beyond what is expected [12, 13]. Prognostication is accordingly very difficult [14, 15]. To deal with uncertainty is a major challenge for physicians working in the neuro intensive care field [16, 17].

The Norwegian Neurological Society has published recommendations concerning disorders of consciousness (DOC) emphasizing the early prognostic uncertainty and long recovery trajectories after neurological injuries [18]. They advocate carefulness in early evaluations of whether to limit or withdraw treatment: “It might be appropriate to consider limitations of LST, but often not before at least after 1 year observation time for traumatic injuries” [18]. In Norway, in contrast to in the UK, no detailed guidelines exist on how to perform withdrawals in relation to neurologically impaired patients lacking capacity to consent [19, 20].

Legal frames for decision-making as regards patients lacking capacity to consent vary between countries: In the US physicians need consent from family to limit LST. In most European countries including Norway the attending physician has final decision-making authority [21, 22]. Very few Norwegians write Advance Directives or in other ways prepare for circumstances involving a sudden loss of decision-making capacity.

This paper is the last part of a three-phase study of sTBI in a level 1 trauma hospital in Norway. The first, a retrospective study based on registry data and medical records found that decisions to limit LST were common, in addition most decisions were made early after admittance and were closely linked to death [1]. The second study where qualitative methodology was used explored experiences and reflections around prognostic doubt in the early phase (> 72 h) after admission [23]. The strategies applied were provision of treatment trials, evaluation of individual treatment responses and multi-disciplinary team discussions prior to the final conclusions. A surprising finding, and contrary to ethical and legal advice, the Norwegian physicians did not seem to give weight to patients’ preferences in the first days after an injury.

In this paper, we seek insights about ethical reasoning and communication strategies among physicians when dealing with uncertainty and doubt around questions about whether to withhold or withdraw life-sustaining treatment in sTBI patients—the subgroup showing minimal or no neurological improvement in the later trauma hospital phase.

## Methods

### Setting and study participants

Oslo University Hospital (OUH) is the largest trauma center in Norway, with a catchment area covering half the Norwegian population. Within the OUH neuro intensive care unit (NICU), severe traumatic brain injured patients are cared for by nurses and a highly specialized treatment team involving neurosurgeons, intensive care physicians and rehabilitation physicians. We interviewed senior consultants from OUH with extensive trauma care experience who were actively engaged in patient care. We recruited participants by e-mail to all senior consultants via the heads of the relevant departments, as well as by individual invitations. Our intention was to offer the possibility to participate to all, but we were eager to recruit the most engaged and experienced ones. By e-mail we provided background information in order to prepare the participants for the interviews. Participants were provided with definitions of futility and PIT, and they were asked to focus on the “PIT cases” which involve a dilemma and thus demand a balancing of different considerations [9]. Semi-structured interviews were conducted with 18 physicians who were affiliated with the NICU: 9 neurosurgeons (N), 7 intensive care physicians (I) and 2 rehabilitation physicians (R). We included all in order to maximize the sample diversity and because all these physicians, regardless of specialty, are main decision-makers with regard to level of treatment. Our focus was not on differences between specialties, but rather on similarities and differences within the group as a whole. Among the 18 interviewed physicians, 7 were women and 11 men. Their mean age was 53 years (range 38–73), and the mean length of experience dealing with sTBI patients was 14 years (range 6–30).

### The interviews

The interviews took place between April and September 2017, during regular working hours, and lasted approximately 1 h each. AR conducted the interviews and transcribed the audiotaped interviews verbatim. The interview guide was developed by the research team; an intensivist (AR), a neurosurgeon (EH) and an ethicist (RF). It was based on the researchers’ expertise, relevant literature and unresolved questions in prior studies by our research group on treatment-limiting decisions in sTBI [23]. The interview guide consisted of so-called items to be covered, see Table 1. A list of items instead of fixed questions enables the researcher to employ the guide in a flexible manner. The interview guide was slightly adjusted along the course of the study, accordant with qualitative methodology [24, 25]. The opening question was: “Please share your experiences and reflections regarding encounters with severely brain injured patients and their families, when you were in doubt about whether to continue, withhold or withdraw life-sustaining treatment. Please use real-life cases you have been involved in. How did you perceive the situation at hand, how did you reason and act?” Open-ended questions were used to access descriptions in the physicians’ own words. Probing was used to further explore the meaning of what was said. According to common understanding in qualitative methodology the data is co-created by the interviewer and the participants in the sense that the researcher’s interviewing skills, attention and sensitivity during the interviews will influence the data collection [24–26].

**Table 1:** Interview guide

Items to be covered	
Strategies you use to deal with uncertainty and doubt Crucial steps in the decision-making process Role/ collaboration within the team Communication with families To estimate and communicate prognosis Weight you give to different considerations The role of patient’s values, wishes, will Impact of family input Timing issues; early vs late withdrawals Concerns about current practice	

### Analysis

After 18 interviews saturation was reached, which means further data collection cease to add understanding in relation to what has already been gained. Qualitative thematic analysis was used, for an example see Table 2. Here, a theme refers to a word, concept or sentence which captures something important in relation to the research question, represents some level of pattern, response or meaning within the data based on the researchers’ judgment. We used the following analytic steps: 1. Reading of interviews for overall impression and searching for preliminary themes (AR, RF), 2. Re-reading, searching for meaning-units and coding of interviews word by word using inductive coding (AR), 3. Looking for similarities and differences, across and nuances within the interviews (AR, RF), 4. In order to understand the content in more depth the researchers decided to further develop the analysis for this paper, with a focus on (1) *what physicians told about patients with prolonged NICU stays who showed no or minimal neurological improvement* and (2) *communication strategies and ethical reasoning applied by physicians in situations related to treatment-limiting decisions when doubt was prevailing* (AR, RF, EH). In the analytic phase, the research team moved back and forth between a position of critical thinking and interpretation of details such as single words or expressions, to a bird’s-eye view and search for the essence of the findings relevant to the research questions. Some qualitative researches use pre-defined theoretical frameworks to structure their analysis. Our analysis was deliberately not constrained by any anchoring in theory. We strived for an open-ended, flexible, empirical data-driven, clinical relevant analysis. NVivo Pro 11 (QSR International, Melbourne, Australia) was used as a tool to organize data and support our analysis [27].

**Table 2:** Steps in an analytic process towards development of themes

Meaning unit	Codes	Theme
“She was treated in our unit for months. I examined her repeatedly. There was only subtle clinical improvement. She began to open her eyes, but did not give contact. We managed to wean her off the ventilator. With regard to her prognosis, what can I say? I really did not know. I thought she was going to be institutionalized for the rest of her life and dependent on others. Hopefully, she would regain some level of consciousness. My judgement was that we should continue life-sustaining treatment, but within limits; not intubate her again and not resuscitate. I definitely thought we should give her rehabilitation. But, to be honest I do not really know if it is worth it; to work hard with rehabilitation for months and years to achieve such an outcome. I do not think there really are any clear answers to these questions.” 9_N	High risk of an unacceptable outcome Uncertainty not resolved Fallibility of judgement Physician´s doubt and moral ambiguity A willingness to discuss TLD despite uncertainties A sense of responsibility for moving processes forward Nuanced decisions Make plan both for a best-case scenario and a worst-case scenario	Proactivity

## Results

A pattern emerged which captures two contrasting strategies and thereby exposes inter-physician variability in end-of-life decision-making in cases where uncertainty and doubt prevail:

- The proactive strategy
- The wait-and-see strategy

### The proactive strategy

One group of physicians described their strategies for mitigating uncertainty and doubt and dealing with challenging ethical dilemmas as proactive. They emphasized their duty to secure that their treatment did not lead to a life unacceptable for the patient e.g. survival to a permanent vegetative state. They initiated discussions, took leadership in clarification and deliberation processes, were open to discuss the issue of treatment limitations in all stages after an injury, but were also humble, and aware of potential fallibility. Nevertheless, they believed that through collaboration within the multidisciplinary team, communication with the patient´s family and sufficient time to evaluate the medical situation, they could reach decisions on whether to continue life-sustaining treatment or whether to consider the withholding or withdrawing of some modalities or all life-sustaining treatments.

“The core issue is: What are we rescuing this patient to? Is continued treatment in the patient´s best interest? Every single patient needs an individualized judgement. I feel I am proactive. I often initiate discussions about level of care. Others do not.” 14_I.

Our study revealed that “best-interest meetings”/”level of care discussions” /discussions about whether to continue, withhold or withdraw life-sustaining treatment were not conducted on a foreseeable or regular basis. Therefore, initiation of discussions was critical. The proactive physicians described how a trigger for such discussions could be their own or others’, including the family’s, doubt about a patient’s best interests. Other triggers could be lack of improvement despite maximal therapeutic effort, neurologic deterioration or repeated problems such as new infections or breathing problems.

“Whenever there is doubt about a patient´s best interests, I think it is appropriate to open up discussions. I believe a proactive approach is very important. To explicitly address and try to gradually resolve the ethical issues that confront us.” 3_N.

“Dr. X initiated discussions. The other physicians that previously had been responsible for her treatment in the neuro-intensive care unit had not made any such initiative.” 3_R.

Many of the proactive physicians talked about the need for nuanced decision-making. They advocated the value of preparing for the worst, but working towards the best possible outcome, e.g. rehabilitation may co-exist with limitations of some life-rescuing or life-sustaining treatment modalities. A DNR order is simply a plan for what to do if a cardiac arrest occurs; the goal of treatment is still best possible functional outcome.

“In some devastating cases I believe the right thing to do is to be proactive, set limits, but provide rehabilitation. To limit certain elements of treatment in case of a future deterioration is a very different decision than to withdraw.” 15_I.

The proactive physicians tried to anticipate future developments, especially with regard to imminent threats but also long-term consequences, and to prepare. In their view, no plans should be seen as static, but rather open to adjustment as the situation evolved.

“The difficult question is for how long should we continue, if the patient fails to respond? I think we need to have an ongoing discussion the first year after the injury. Re-evaluate regularly.” 12_R.

The proactive physicians, regardless of specialty, considered dialogue with the family as a crucial way to build a common situational understanding and as an integrated part of the physicians’ professional role.

“We talk with families about the situation at hand, about what can be expected and the uncertainty of our prognostication. By having repeated talks, we develop a relationship and can gradually build common ground. In my experience to develop this common situational understanding with families is usually possible, although with some families it just isn´t.” 3_N.

The proactive physicians described how they tailor communication, pay attention to emotional aspects in the team-family interactions and try to contribute to empowerment. The process involves gradually puzzling the pieces together, making sense of conflicting facts or signs and striving to find a coherent story.

“I tell families; this is a process, we will walk with you step by step.” 1_I.

Some physicians described personal techniques when approaching and supporting families.

“I think they need an understanding about what really happened. I use a technique when I talk with the family: I go back. I go back to search for the story. I try to walk them through what happened. Try to puzzle the pieces together one by one.” 4_N.

They accentuated the importance of listening skills and good verbal as well as non-verbal—and individualized—communication.

“I try to evaluate what the families are able to absorb and what they are interested in. I try to be selective. What matters most for the family right now? What is the most important thing I would like to address today? I think they need to develop their own concepts, be able to retell the story with their own words. I use a kind of challenge and response technique. If I understand that they are not able to absorb more I focus on working on a relational level.” 7_I.

### The “wait-and-see” strategy

The decision-making processes among the ones who preferred to wait and see were dominated by prognostic uncertainty and their commitment to avoid any self-fulfilling prophecies, with focus on not to withdraw too early. In circumstances of doubt they emphasized their duty to treat. Especially with young patients, they found even discussions of treatment limitations to be inappropriate.

“Treatment limitations are not an issue we discuss in relation to young patients, unless we approach a situation of possible brain death” 1_I.

Professional experiences seemed to influence their strategy. They shared stories of patients who had obtained a quality of life far beyond what anyone, including themselves, had ever expected during the acute phase.

They considered just a small chance of improvement sufficient in order to continue aggressive treatment. Neurologically injured patients need months and sometimes years to reveal their true recovery potential, they argued. Given the level of uncertainty concerning long-term outcome, they perceived a focus on short or intermediate goals to be more appropriate. Why rush? Who am I to make decisions to limit or withdraw treatment in this phase?

“We must admit that our ability to be sure about whether a patient will regain consciousness or not is limited.” 16_I.

These physicians believed that it was respectful to wait, let the consequences of the injury gradually unfold, remain vague and simply accept the uncertainty of the situation, rather than to force a premature resolution or prepare for all kinds of different scenarios.

“If we need to consider the issue of death and dying the discussions occur late, if they occur at all.” 5_N.

In cases with physiological instability, the postponing of sensitive discussions and absence of plans for potential deteriorating was problematic as staff working night shifts who did not know the patient might have to make decisions based on insufficient knowledge.

“When the patient was transferred from the intensive care to the intermediate rehabilitation ward it was unclear what we were expected to do if we were faced with a new deterioration. The injury was devastating, with no signs of significant clinical improvement. The patient was weaned off the ventilator and was given nutrition via a PEG, but he was not reliably stable with regard to his airway and breathing. There was a risk he would need a new intubation, but was a re-escalation of life-sustaining treatment really in this patient´s interest? No explicit plan existed.” 2_R.

In some cases the wait-and-see physicians experienced moral ambiguity, but without sharing their thoughts with colleagues or the patient’s family. They did not allow themselves to act on, or even communicate their doubt.

“Even though I worried about the patient´s outcome, I could not allow myself to think about treatment-limitations” 8_I.

Moreover, the physicians felt that they could only take responsibility for a limited part of the complex treatment chain. They emphasized their lack of knowledge about what happened to their own patients after discharge and were uncomfortable with giving advice to colleagues responsible for the next chain of treatment.

“I am responsible for the patient in the neuro intensive care phase. How patients are dealt with in the rehabilitation phase is beyond my expertise. I work toward stabilizing the patient, work towards giving him or her access to specialized rehabilitation. What happens after discharge from the neuro intensive care unit is not my responsibility.” 14_I.

Like the proactive, the wait-and-see physicians were committed to support the patients’ families, however by different means. Both groups described a long path where common ground was built through multiple conversations over time. They believed there was not a need for the same directness now as early after admittance, where death nearly always was mentioned to the families as a possible outcome. When the situation somehow stabilized, they preferred to stop talking about death and dying.

Situational understanding evolves over time. The wait-and-see physicians described how the doubt about the patient’s best interest sometimes paradoxically seemed to increase along the treatment trajectory. Emotional bonds to the family members grew stronger with time.

According to the wait-and-see physicians, the family’s resistance to address “what if” could be the main reason why they were not able to move forward. This sensitivity towards the emotional state of the family partly guided their choices. They chose to delay the process if they felt that the family was not ready, while the proactive believed in their ability to move forward through repeated communication, support and guiding.

“What I find really difficult is the families that hold on to very unrealistic expectations. Some families are just not able to listen or absorb what we are trying to tell them. If e.g. I want to address the issue of what to do if the situation deteriorated even further, but sense I cannot reach the family, I will be afraid to damage the relationship and choose to keep focus on here and now.” 3_N.

## Discussion

TBI patients with ongoing LST showing no or minimal signs of neurological improvement in the later trauma hospital phase trigger feelings of doubt and moral ambiguity. We found practice variations with regard to both whether and when physicians initiate team discussions about limitations of LST, and how much physicians involve the family in order to determine a patients’ best interests. This has important ethical implications.

### Long term prognostic uncertainty hinders resolution processes

Even though in some cases more time means greater prognostic certainty, in other cases prognostic clarity does not emerge within the weeks and months of a NICU stay, and best-interest discussions and decisions may be postponed to an ever-more-distant future [28].

Pier et al. found a divide between physicians and nurses as regards their views on major impediments for a team’s ability to identify potentially inappropriate treatment and prevent overtreatment [29]. Physicians often explained their avoidance by referring to prognostic uncertainty, while nurses perceived that poor communication and physicians’ unwillingness to address end-of-life issues were reasons behind overtreatment [29].

### Proactive or wait-and-see strategies

Our physicians were all aware of the ethical complexity of the situation and the emotional stress the family members were in, but our study reveals how ethical awareness does not necessarily lead to ethical reflection within the team or openness towards the family. The threshold for when the physicians open up to an ethical discussion concerning the limitation of life-sustaining treatment varies. We identified two strategies or ways of acting and interacting based on goals, values and believes: The proactive and the wait-and-see.

We believe the reasons behind the two emerging strategies are multifaceted and beyond the scope of our study to fully explain [30–32]. Nevertheless, it seems like the wait-and-see strategy is anchored in a deep sense of humbleness towards the long recovery paths of neurological injuries, a non-judgmental attitude to quality of life after severe brain injury, and a sense of duty to ensure that all has been done to secure a best possible outcome. To this adds that most physicians have experienced recovery among patients assessed to be beyond rescue. They believe vagueness in prognostic language is actually helpful because it captures what can be said about what to expect [33]. To give advice and intervene faced with uncertainty is sometimes inappropriate or may not be the most helpful response. These physicians give weight to the acceptance of uncertainties and the value of giving space for a process to gradually unfold. The proactive physicians, on the other hand, put more weight on their responsibility to shape processes, not merely react to what is unfolding. They are open to consider different options—although they are also aware of prognostic uncertainty and the possibility of fallibility. In our opinion there are unanswered questions and underlying hidden issues behind these differences. Physicians do not always reveal what guides their ethical reasoning. What receives attention and is given most weight varies. Self-awareness and open ethical discussions are necessary [34]. Are the proactive sufficiently concerned about avoiding discrimination and protecting vulnerable patients and families? How is their proactivity appreciated by the family? Is it perceived as helpful or as a threat? The wait-and-see physicians’ tendency to avoid sharing their moral doubt with the team and family members prevents open ethical discussion about the patient’s best interests.

Even though agreement among the involved parts makes decision-making easier, some have focused on how it is important to disagree about end-of-life decisions [35]. Divergent views and strategies reflect the complexity of the issue. In the end, however, it is not the physicians’ own values and preferences, but the patient’s wishes and/or best interest that should be the guiding principle for person-tailored preference-sensitive choices.

### A good ethical climate

We consider it a strength that physicians within one institution hold different views and modes of thinking. These modes are complementary. Physicians may challenge and influence each other and hopefully make more balanced decisions, given there is a culture for transparent processes, team-based approaches and daily inter-professional close collaboration [36]. Our physicians described, despite the here exposed inter-physician variability, the well-developed approaches of multi-disciplinary teamwork as one of the strengths of the climate they worked within. This should also include ethics.

Van den Bulcke et al. define a good ethical climate as one in which there is a culture of ethical awareness, self-reflective and empowering leadership by physicians, open interdisciplinary reflection, mutual respect within the teams involving an acceptance towards different views, active involvement by all members of the team and not avoiding E-o-L decisions [34].

### Patient’s preferences

The Choosing Wisely Campaign is in line with the Norwegian ethics guidelines on limitation of life-prolonging treatment. Both advocate a presentation of alternative options to patients or family from early on and a clarification of the patient’s preferences. The very first paragraph in the Norwegian guidelines suggests the use of treatment trials whenever there is doubt about the benefit or prognosis, and to be explicit to the family about the possibility of a subsequent withdrawal if the treatment goals are not obtainable [22]. Openness, preparation and clarification of goals are important along this line of thinking which may create trust and contribute to the reduction of unrealistic hope among family members.

In our prior work on the first 72 h it was revealed that Norwegian NICU physicians did not elicit or give weight to patient’s preferences [23]. This leads us to the question: If not during the first 72 h, then when? With patients who are treated for weeks or sometimes months in the NICU there are certainly opportunities and time for dialogue, but according to our findings even in the later phases of the trauma hospital stay the patient’s preferences are seldom referred to as the primary reason behind the decision whether to continue, limit or withdraw. One explanation may be that it is simply too hard to know, reconstruct or interpret the patient’s preferences if the issues of how to respond to different situations have not been raised prior to injury. Another explanation is that physicians sometimes avoid hard conversations and thereby miss the opportunity to get helpful guidance from families.

### Align with families

No families are alike. Neither do families always act as one entity. Our study revealed how physicians often have their individual way of approaching and involving families. Some are more explicit in their communication, they explain words and concepts for the family e.g. “treatment trail”, “life-sustaining treatment”, “what a withdrawal process entails” or “how you and your loved one will be supported through a process of withdrawal/dying process” or ensure families have understood “what lies behind decisions to continue or to withdraw”.

Discordance between doctors and families as for their understanding of prognosis is recognized as one barrier for building common ground crucial for resolution processes [37–39]. Adding to the complexity is the fact that some families may seek involvement in and impact on decisions regarding their loved ones from an early stage after the injury [40]. Other families appreciate all time given and some have according to a Norwegian interview study even voiced a feeling of anger towards what they perceived as insensitive physicians in the trauma hospital being too pessimistic about prognosis and initiating discussions on the issue of limitations far too early [41]. It is crucial for the teams to strive to develop an understanding about what individual families find helpful in order to promote families’ well-being and sense of agency [42].

### Establish guidelines for late withdrawals

Is there reason to fear that LST in some cases will be continued indefinitely without a shared understanding of its justification, but just as the default [43–45]? A robust system which offers good support in cases of withdrawal in the phases after discharge from the trauma hospitals, e.g. a vegetative patient in a nursing home or local hospital setting, can prevent a sense of rush and support a delay of actions promoted by wait-and-see strategies in the trauma hospital [19, 20, 46]. In Norway, specialized neuro-palliative services are poorly developed. In UK on the other hand, they have recognized how difficult late withdrawals are and crafted detailed guidelines to support staff, families and secure the right of patients to avoid prolongation of treatment no longer in their best interest [19]. In USA neuro-palliative care is a growing field developed as a response to unmet patient and family needs [47].

### Ethics support services are rarely used

To determine a patient’s best interests may be difficult. Such an evaluation involves estimating and communicating what to expect, but also difficult value issues which may give rise to conflicts within the team or with the family. Disagreements and conflicts can be resolved through dialogue and by giving a situation time, furthermore by second opinion of external medical experts or involvement of clinical ethics services. All Norwegian hospitals have a clinical ethical committee (CEC) and Norwegian doctors find CEC discussions useful [48]. Our interviews reveal that involvement of a clinical ethics committee (CEC) is not among the mentioned strategies for resolving doubt among the neuro intensive care physicians. This is thought-provoking as sTBI cases involve ethically challenging patients. It is worth asking why such a service is not applied in this setting. CEC is meant to be a support in the making of decisions also to secure patient rights, especially since advance directives are so rare in Norway. We believe the ethical issue of protecting the rights of persons lacking capacity needs more focus.

### Limitations

Only physicians were interviewed in our study. Nurses, family and patients´ perspectives are also needed to obtain a richer and more complete picture of the issues here discussed. Our study focuses narrowly on the ethically most challenging cases. The primary investigator was familiar with some of the mentioned cases as well as colleagues and the hospital culture through her clinical work as an intensivist. We believe the familiarity between participants and the interviewer fostered honest and open reflections. However, closeness to the research topic can produce bias; therefore, the last author, who is not employed in clinical practice, but is an ethicist familiar with the value issues in end-of-life decision-making, actively participated in all steps throughout the research process—in particular the development of the interview guide and the interpretation of all the interviews. We have only studied physicians in one (albeit large) hospital. This may be seen as a limitation. However, we believe some of our points can be transferable to physician groups responsible for brain trauma care in other care contexts and countries. The categorization of professionals into groups here presented will never reflect the whole truth. The same physician may be supportive of the wait-and-see side in one situation, while another may call for more proactivity.

## Conclusions

Depending on the individual physician in charge, ethical issues may rest unresolved or not addressed in the later trauma hospital phase. Nevertheless, team collaboration serves to mitigate inter-physician variability. There are problems and pitfalls to be aware of related to both proactive and wait-and-see approaches. The timing of best-interest discussions and treatment-limiting decisions remain challenging after severe traumatic brain injury. Routines for timely and open discussions with families about the range of ethically reasonable options need to be strengthened.

## Data Availability

The approval from the Institutional Data Protection Officer did not include free sharing of data.

## Abbreviations

DBI: Devastating brain injury
DOC: Disorders of consciousness
LST: Life sustaining treatment
OUH: Oslo University Hospital
PIT: Potentially inappropriate treatment
sTBI: Severe traumatic brain injury
